# Unmet Needs of Children and Young Adults With ADHD: Insights From Key Stakeholders on Priorities for Stigma Reduction

**DOI:** 10.1177/10870547241297876

**Published:** 2024-11-15

**Authors:** Marlies Jolinde Visser, Ruth Maria Hendrika Peters, Marjolein Luman

**Affiliations:** 1 Athena Institute, Faculty of Science, Vrije Universiteit, Amsterdam, The Netherlands; 2 Amsterdam Public Health Research Institute, The Netherlands; 3 Department of Clinical, Neuro and Developmental Psychology, Faculty of Behavioural and Movement Sciences, Vrije Universiteit, Amsterdam, The Netherlands

**Keywords:** ADHD, stigma, stigma reduction interventions

## Abstract

**Introduction:**

Individuals with ADHD continue to face stigma, which can negatively impact their access to, adherence to, and quality of mental health services, as well as their overall wellbeing. Perspectives of various stakeholders regarding priorities for stigma reduction remain underexplored. This study explores young adult, parent, teacher, and mental health care professional perspectives on unmet needs of children and young adults with ADHD in the Netherlands, in the context of stigma reduction.

**Method:**

A total of 24 respondents participated in seven small focus group discussions (FGDs). The FGDs facilitated in-depth discussions exploring stakeholder group perspectives on unmet needs of children and young adults with ADHD in educational, professional, and social settings. Data was analyzed using thematic content analysis.

**Results:**

Three thematic categories of unmet needs were identified: (1) a lack of awareness, knowledge, and understanding of ADHD; (2) insufficient personalized approaches in education and health care; and (3) limited accessibility of information and support services. Within theme 1, respondents primarily highlighted the importance of improving education for primary school teachers and mental health care professionals. Theme 2 underscored the need for increased capacity for personalization and attention to the sociopsychological factors of ADHD, alongside recognizing strengths. Theme 3 emphasized the need for easy access to reliable information and mental health care, including peer networks, as well as proper follow-up and continuity of care.

**Discussion and Conclusion:**

Findings highlight the need for improving our understanding of ADHD as a complex biopsychosocial condition, which requires specific adjustments in education and mental health care. Interventions to improve support and disrupt stigmatization should meet diverse needs, incorporate multi-level strategies, and involve key stakeholders.

ADHD is a common neurodevelopmental disorder characterized by persistent levels of inattention and/or hyperactivity and impulsivity (American Psychiatric Association, 2013). Due to the prevalent negative attributions and misconceptions associated with ADHD, individuals with ADHD are at risk of encountering public and internalized stigma (Bisset et al., 2021; Lebowitz, 2013; Mueller et al., 2012; Speerforck et al., 2019; Visser et al., 2024). Mental health stigma continues to have adverse impacts on social and health outcomes (Thornicroft et al., 2022), and has been conceptualized to comprise; stereotypes (cognitive responses), prejudice (affective responses), and discrimination (behavioral responses; Corrigan et al., 2005). Public stigma refers to a process where negative stereotypes are endorsed leading to discrimination by the general public (Corrigan et al., 2005). For example, one study found that university students in the United States (U.S.) showed significantly less desire to engage with individuals with ADHD, compared to their peers without ADHD (Canu et al., 2008). Public stigma may evoke internalized stigma among individuals with ADHD or their family, referring to internalization of public prejudice and discrimination related to ADHD (Mueller et al., 2012). For example, in a qualitative study including parents of adolescents with ADHD in the U.S., parents noted that ADHD-related stigma affected the self-confidence of their children (Koro-Ljungberg & Bussing, 2009). Further, our previous study found persistent overt and more subtle expressions of ADHD-related stigma, and suggests these to negatively impact individuals with ADHD in the Netherlands. Our findings revealed nuanced gender differences in the characterization of individual manifestations of ADHD-related stigma. Young adult women typically emphasized altered identity development, masking, and loneliness, while young adult men typically highlighted internalized negative attributions, nondisclosure, and reduced help-seeking behaviors (Visser et al., 2024). Finally, public and self-stigma can negatively impact (mental) health and wellbeing as it can affect help-seeking behaviors, treatment adherence, and self-esteem of individuals with ADHD (Hinshaw et al., 2006; Mueller et al., 2012).

Reducing mental health stigma is complex and challenging, given the various structural and sociocultural factors shaping stigma and its multifaceted nature and implications. Researchers have established that stigma operates across socioecological levels, including individual, interpersonal, community, and structural levels, which delineate the target scope of interventions aimed at stigma reduction (Cook et al., 2014; Heijnders & Van der Meij, 2006). A growing body of literature explores mental health stigma reduction across these levels and focuses on strategies such as protest, education, and contact, or analyses the effectivity of approaches to reduce mental health stigma across various contexts (Corrigan & Shapiro, 2010; Dalky, 2012; Gronholm et al., 2017; Jorm, 2020; McCullock & Scrivano, 2023; Mehta et al., 2015; Thornicroft et al., 2015, 2022). In their meta-review of meta-analyses, McCullock and Scrivano (2023) found that education and contact were most often employed to reduce public mental health stigma, while psychosocial interventions were commonly employed for self-stigma reduction. They found that contact and education strategies showed small-to-moderate effects on stigmatizing attitudes and behavioral intentions. However, the literature on mental health stigma reduction remains inconclusive as studies present heterogeneous results regarding the effectiveness of specific strategies, the impact on the long versus short term, impact on various types of stigma, and/or on different target groups. Moreover, researchers have cautioned for unintended consequences of anti-stigma efforts, if not carefully considering effectiveness of interventions employed (Corrigan, 2016).

Researchers and mental health care professionals have called for various approaches to reduce ADHD-related stigma. In their review of the literature, Bisset et al. (2021) conclude that there is a need for efforts increasing ADHD-related mental health literacy, improving recognition and attitudes toward ADHD. A European consensus statement by researchers and mental health care professionals from 28 countries suggests strengthening professional education by emphasizing a lifespan perspective, diagnostic assessment, and treatment for ADHD for professionals in psychiatry and students in (mental) health (Kooij et al., 2019). Further, researchers have called for the engagement of families and individuals with lived experiences—those directly impacted by ADHD—in the design of interventions aimed at reducing ADHD-related stigma, based on qualitative research conducted in the U.S. (Koro-Ljungberg & Bussing, 2009). Moreover, recent studies have emphasized the importance of considering social norms and gender roles in understanding and addressing ADHD-related stigma, as well as the need to tailor stigma reduction approaches to individual or group needs (Canu et al., 2023; Visser et al., 2024).

Additionally, it is important to incorporate the perspectives of individuals with lived experiences into research to inform the design of effective interventions aimed at reducing ADHD-related stigma. This strategy is increasingly recognized as a priority for improving research processes and mental health care, and for reducing stigma (Jørgensen & Rendtorff, 2018; Kilbourne et al., 2018; Laitila et al., 2018; Sonuga-Barke et al., 2024; Stuart & Sartorius, 2017). In the past, mental health stigma reduction efforts were traditionally designed by mental health care professionals or groups with psychiatric expertise (Gronholm et al., 2017). These approaches faced criticism for potentially concealing objectives to promote a positive image of psychiatric services and mental health service use, for emphasizing the biogenetic etiology of mental illness, alongside concerns about the potential contribution of mental health professionals to stigma (Ashton et al., 2018; Charles, 2013; Corrigan, 2016; Pilgrim & Rogers, 2005; Schulze, 2007). A growing number of studies concurrently employ qualitative stakeholder inquiry to inform interventions (Corbière et al., 2012; Shahwan et al., 2022). Various studies on children, adult, parent, and professional perspectives on ADHD and mental health care have been conducted (Bailey & Simpson, 2008; Carr-Fanning & McGuckin, 2018; Gallichan & Curle, 2008; Hughes, 2007; Schrevel, Dedding, & Broerse, 2016; Schrevel, Dedding, et al., 2016). However, no study has previously explored multiple stakeholder perspectives to set priorities for ADHD-related stigma reduction specifically. To address this gap, this study builds upon a related study exploring ADHD-related stigma and the role of gender (Visser et al., 2024) and aims to get insight into the unmet needs of children and young adults with ADHD and to identify priorities for improving support and reducing ADHD-related stigma by exploring young adult, parent, teacher, and mental health care professional perspectives in the Netherlands.

## Methods

### Sample and Recruitment

Detailed recruitment and sampling strategies of this study have been described elsewhere (Visser et al., 2024), but in short, participants were recruited for focus group discussions (FGDs) organized between December 2022 and July 2023. We used convenience sampling through advertisements on social media pages, the website of an ADHD interest group and a parent organization. In addition, the professional and social networks of the involved researchers were used. A total of 24 respondents participated in a total of seven FGDs. Young adults aged 18-30 with a self-reported recent or past ADHD diagnosis were eligible for inclusion. We did not confirm their diagnosis (i.e., we did not let our respondents fill out an ADHD questionnaire). However, the young adults all mentioned during the FGDs they were diagnosed in a Dutch mental health clinic recently or when they were younger. There were no further exclusion criteria (e.g., other classifications or the use of medication). Caregivers were eligible if they self-reported to have one or more child(ren) with ADHD diagnosed at primary school age (aged between 6 and 12 years) and primary school teachers and mental health care professionals were eligible when self-reporting to have professional experience with individuals with ADHD.

### Focus Group Discussions

We used FGDs to explore diverse perspectives and allow interaction between respondents to co-construct and co-define responses (D. L. Morgan, 1997). Each group participated in a small group-specific FGD, and young adults participated in same-sex FGDs. We chose single-sex FGDs for young adults since we were interested in potential gender differences between perspectives on unmet needs and priorities for stigma reduction. We define a small FGD as a group of three to five participants. We chose this approach to allow in-depth discussion and valuable contribution of all respondents to share experiences (Toner, 2009). The FGDs with young adults were organized face-to-face while the other stakeholder groups participated in online FGDs. While all FGDs employed multiple thematic discussions on different stigma-related themes taking 1.5 up to 3 hr in total, this paper presents findings from the discussion on ADHD-related stigma reduction only. This discussion took between 30 and 60 min with each stakeholder group. We asked respondents open questions regarding (1) their perspectives on how different stakeholders (i.e., individuals with ADHD, (mental) health care professionals, teachers, family, and social networks) can best support the reduction negative stereotypes, prejudice in discrimination in various contexts including educational, professional, social, or family contexts, and (2) what various stakeholders can do to better support children and young adults with ADHD (see Supplemental Material 1). While primary school teachers and mental health care professionals were specifically asked for input regarding their respective professional contexts, young adults and parents were free to share their perspectives pertaining to any context. First, respondents wrote down their perspectives individually, using key words on (online) sticky notes. Their inputs were subsequently discussed in plenary and all sticky notes were included on (online) flipcharts.

### Data Analysis

FGDs were audio recorded and transcribed verbatim with support from research assistants. The first author re-read the transcripts to familiarize herself with the data. The transcripts were coded and analyzed using Atlas.ti 22 and Excel. All coding was performed by MV and discussed with co-authors. Data was analyzed using thematic content analysis (Braun & Clarke, 2006). First, perspectives on support needs and stigma reduction were coded using deductive axial coding on the group level. Subcodes were added during this process when relevant. Second, quote excerpts for each stakeholder group and per key context were extracted and reviewed using Excel spreadsheets. In this step, the first author created subgroups of quote extracts and added additional labels (i.e., subcodes) to identify key subthemes per context and to identify cross-cutting themes. The subcodes that emerged through the process of selective coding characterize the unmet needs as presented in the results section. The perspectives from young adults were prioritized for identifying the key thematic categories. Subsequently, the recurring unmet needs under each theme were based on perspectives of all stakeholder groups. For the purpose of this article, the quotes included in this article were translated from Dutch to English.

### Procedure and Ethics

Before participating in the FGD, respondents completed a short demographics survey and informed consent form. The FGDs with young adults were conducted in Dutch and organized face-to-face at the Vrije Universiteit Amsterdam. The FGDs with caregivers, teachers and mental health care professionals were conducted online. All data were stored on a password protected university drive. Only young adults received financial compensation for their participation in the study and their travel costs since their FGDs were organized face-to-face. After participation, the amount of compensation (100 EUR) was communicated. Ethical approval for this study was granted by the Scientific and Ethical Review Board of the Faculty of Behavioral and Movement Sciences of the Vrije Universiteit Amsterdam, reference number VCWE-2022-131.

## Results

### Respondent Characteristics

A total of 14 young adults participated in four sex-specific FGDs, of which 8 female and 6 male respondents, between the ages of 19 and 30 years with mean ages of 26.2 years (standard deviation (*SD*) = 3.5) and 25.8 years (*SD* = 4.0), respectively (see Table 1). Three teachers within primary education (all women) and four mental health professionals with experience in assessment and treatment of ADHD (one man/three women) participated, each group in one FGD. The last FGD included three mothers, each of whom is raising one or more children diagnosed with ADHD at primary school age, totaling seven children, including two girls and five boys.

**Table 1.** Respondent Characteristics.

Stakeholder groups	Number (*n* = 24)	Mean age (*SD*)	Mean age diagnosis (*SD*)	ADHD presentation (C/I/H-I)	Highest educational degree obtained (HS/V/P/U)	Professional experience with ADHD in years (*SD*)
1. Young adult women (2 FGDs)	8	25.8 (4.0)	23.8 (4.4)	5/3/0	2/0/1/5	—
2. Young adult men (2 FGDs)	6	26.2 (3.5)	19.8 (5.3)	5/1/0	2/1/0/3	—
3. Caregivers (1 FGD)	3^a^	43.0 (5.1)	7.1 (2.8)	6/1/0	—	—
4. Primary school teachers (1 FGD)	3	46.3 (6.5)	—	—	—	14.7 (7.8)
5. Mental health care professionals (1 FGD)	4^b^	45 (20.9)	—	—	—	<18 years: 14.8 (9.1) >18 years: 12.5 (7.5)

*Note.* This table is similar to Table 1 in (Visser et al., 2024). *Note.* FGD = Focus Group Discussion; SD = Standard Deviation; C = Combined; I = Inattentive; H-I = Hyperactive Impulsive; HS = High school; V = Vocational Education; P = Professional Education; U = University Education.

a Participants were all mothers with in total seven children diagnosed with ADHD in childhood (<12 years old), including two girls and five boys.

b Three of the respondents had professional experience with children with ADHD and two of the respondents (also) had experience with young adults with ADHD.

The analysis of our findings revealed three key thematic categories of unmet needs: (1) awareness, knowledge, and understanding of ADHD; (2) personalized approaches in education and mental health care; and (3) accessible information and support services. The perspectives of stakeholder groups on unmet needs of children and young adults with ADHD will be presented below each of these categories (See Table 2). Among young adults, some perspectives arose exclusively from discussions within one sex-specific FGD. However, upon analysis, sex-specific findings did not occur frequently enough to justify presenting the results separately for women and men. Key results are thus presented collectively in the subsequent sections, while highlighting instances when a particular respondent group raised a noteworthy perspective. All respondents and each supporting quote received a number. For example, Q1 refers to quote 1 and R1 refers to respondent 1. An overview of all supporting quotes including the original Dutch quotations is available in Supplemental Material 2.

**Table 2.** Overview of Thematic Categories of Priority for Reducing ADHD-related Stigma.

Thematic categories	Unmet needs reported by stakeholders	Key supporting quotes
Theme 1. Awareness, knowledge, and understanding of ADHD	Integration of information on neurodiversity in education	1–4
	Education for primary school teachers, specifically on recognizing and supporting children with ADHD in their classroom	5–6
	Training for (mental) health care professionals, specifically on recognizing and (monitoring) treatment for ADHD	7–9
Theme 2. Personalized approaches in education and mental health care	Strengthening personalized approaches in education and mental health care, emphasizing psychosocial factors, strengths, and individual differences	10–13, 15–21
	Increasing capacity in primary and secondary school to address individual needs	14
Theme 3. Accessible information and support services	Accessibility of reliable and trustworthy information sources	22
	Easy access to mental health care	23
	Proper psychoeducation, follow-up, and continuity in mental health care	24–27
	Accessible (informal) peer networks	28–31

### Theme 1. Awareness, Knowledge, and Understanding of ADHD

Our previous and related study showed that the majority of respondents identified a dominancy of negative stereotypes and prejudice related to ADHD in society (e.g., “all individuals with ADHD are hyperactive” or “ADHD is a fashion phenomenon”; see Visser et al., 2024). In the current study, young adults observed a general lack of awareness, knowledge and understanding of ADHD and perceived the negative stereotypes and prejudice to translate into discriminatory practices in education and health care (quote 1–2). All stakeholder groups identified a lack of information related to ADHD or neurodiversity across primary and secondary education (quote 3–4). Importantly, young adults stated that this education should prioritize diversity more broadly, rather than highlighting ADHD as different, or excessively highlighting neurodiversity. This approach was thought to promote greater awareness without promoting special treatment.

I think there are opportunities to address it in a broad and subtle way. I mean we shouldn’t announce neurodiversity with great emphasis now. Then it misses the mark . . . then you get that special treatment again. (Q4, R9, Men FGD 1)

Young adults, parents, and teachers reflected on a lack of knowledge of ADHD among educational professionals and a lack of capacity to recognize signs of ADHD and support children with ADHD (quote 5–6). Consequently, the majority of respondents identified a need to better integrate information on ADHD or neurodiversity in education. Further, professional education for primary school teachers was deemed to need strengthening.

They [teachers] don’t know how to help you, that’s for sure. They may know that you have [ADHD], but then they can’t do much with it, so they think, well, never mind. (Q6, R6, Women FGD 2)

Young adults and mental health care professionals identified a lack of knowledge and understanding of ADHD among (mental) health care professionals. Mental health care professionals expressed frustration regarding the limited understanding of ADHD among general practitioners (GPs) and mental health care workers, including psychologists and psychiatrists. They recognized hesitancy and insecurity among colleagues when discussing a diagnostic trajectory or treatment options for those with (suspected) ADHD, and ADHD is often not prioritized in treatment plans due to an initial focus on other classifications (e.g., anxiety or addiction; quote 7–9).

It’s also a bit black and white. You either know a lot about ADHD or you know nothing about it. So, some people [colleagues in mental health care] really shy away from it, because they don’t really know much about it. While it is actually something that you as a practitioner simply need to have knowledge of. (Q8, R21, Mental health care professionals)

### Theme 2. Personalized Approaches in Education and Mental Health Care

Young adults, parents, and teachers recognized a need for more personalized approaches in education, based on previous experiences with a one-size-fits-all approach and discrimination. For example, two young adult men reflected on their experience of doing well in school because they were intelligent. However, due to a lack of personalized educational approaches, they were never challenged and/or never learned how to study and deal with their ADHD (quote 10). Further, young adults described that some well-intended but unhelpful accommodations in education may highlight differences, which was thought to facilitate exclusion and stigma. One example raised involved the provision of additional time for tests in education, which was perceived as labeling individuals due to differences, evoking exclusion (quote 11). Parents added that a personalized approach depended on the teacher. They reported that it was challenging to get some teachers to take their child’s ADHD diagnosis seriously, especially when children are well adjusted to their medication and/or do well in school (quote 12). Young adults also mentioned instances of discrimination related to their ADHD. For example, one young adult man reflected on his past experiences with exclusion from educational activities, to prevent him from distracting other children in the class (quote 13).

I had a desk in the team room. Where I could sit every day. Instead of trying to make me sit in class. I had to sit alone in a room. . . . They thought ‘he better sit there, he can’t distract anyone that way’. They just pushed me away. (Q13, R12, Men FGD 2)

Teachers acknowledged challenges with a personalized approach and with supporting children with ADHD in classrooms, specifically due to the lack of capacity at their school, full curricula, and heavy workloads. They expressed a need for specialist support, such as advice from a behavioral specialist on how to adequately manage and guide children with ADHD (quote 14).

I: “Is there room for this [differentiation] in education?”

R23: “I don’t think much.”

R22: “Still insufficient.”

R23: “Because you have so many goals that need to be achieved, so many lessons that need to be crossed off, and there is never enough time. . .”

R22: “Perhaps that is not so much the task of the teacher alone. . . . Now it’s really just a child with ADHD who just goes to class with the diagnosis, with or without medication, and the teacher has to discover without background, without extra training, without anything from the PABO^1^ how to manage such a student with ADHD. Can you expect that from a teacher with 29 other [children]? Doesn’t that actually require a [behavioral] specialist who also helps a child with that?” (Q14, R22 & R23, Teachers)

In the context of mental health care, young adults expressed the need to strengthen personalized approaches based on various unmet needs in mental health care. For instance, some young adults reflected on not feeling understood by health providers involved in ADHD care (quote 15). Other unmet needs during appointments with mental health care professionals included a desire for more in-depth conversations that consider one’s individuality and personality (quote 16), increased involvement of partners or other family members, and the incorporation of discussions about strengths, identity, and how one relates to others and society. Young adults stated that currently, the focus of their appointments mainly revolves around managing their symptoms, often through medication or cognitive behavioral therapy, for example (quote 17–18). Emphasizing strengths was seen as a potential way to prevent the negative impact of ADHD-related stigma and promote a more positive self-concept (quote 19–21).

I really missed that community aspect or that social aspect of ADHD in my therapy. Not that therapy was bad, it really helped me a lot, but it was very much about me as an individual and what I can improve. I myself am convinced that it is not just a disorder, but also something in relation to society. I actually really miss the conversation about what it means to be a neurodivergent person in that world. (Q17, R8, Women FGD 2)

Especially stimulate one’s strengths. So, approach someone with ADHD positively, and when someone does something well, really mention it. Instead of just emphasising the negative. Because if you have ADHD, you sometimes already have the idea that you are different from others, but if you emphasise the positive, then someone can get a good feeling. And that’s something I really missed. (Q19, R11, Men FGD 1)

### Theme 3. Accessible Information and Support Services

Several factors were identified as hindering health-seeking behavior and timely access to appropriate support services for ADHD. First, mental health care professionals noted the prevalence of polarizing messaging about ADHD in the (online) media and highlight the challenge of finding trustworthy sources. Additionally, debates between professionals with differing perspectives on ADHD, along with conflicting information, were found to exacerbate misunderstandings and impede individuals from accessing reliable and comprehensive information about ADHD timely (quote 22).

It is so difficult that people [the general public] can no longer see the wood for the trees. That there is so much information and that no one really knows where [to find it]. What is really reliable? That views sometimes differ. . . . I don’t think that a lot of new sources have to be made available per se. But the reliable sources must be easier to find. That you have a place where people know, okay, I can go there. (Q22, R20, Mental health professionals)

Second, young adult men in particular described that ADHD-related stigma (see Visser et al., 2024) and stigma associated with mental health care services act as barriers preventing individuals from seeking care. Moreover, due to the current reality of long waitlists to access mental health services, individuals do not receive care in a timely manner. This was found to hamper one’s ability to check-in for low-level support, for instance regarding managing ADHD in daily life. Young adults and mental health care professionals therefore identified the need for low-threshold and accessible health care options to encourage help-seeking and ensure timely and easy access to mental health services. Providing accessible treatment options or support outside of mental health care, such as through a referral from the GP regardless of ADHD diagnosis, may help reduce barriers to seeking care by circumventing perceived stigma (quote 23).

R9: “Preventing people from avoiding care, and using positive references. . . “

R10: The diagnosis policy of ADHD. For many people. . . from a GP to mental health services is quite a step, because mental health services are kind of seen as the remnants of the madhouse. . .”

R11: “Yes, you only go there when there is really something wrong.”

R10: “At the same time there are also GPs who . . . can help me with a referral. . . .Without the label, help is being provided. That help often has an ADHD direction, but is . . . not necessarily based on a diagnosis. That would reduce the barrier for many people. Someone who is studying with me, for her that was the reason to feel free to go.” (Q23, R9, R10 & R11, Men FGD 1)

Third, several young adults expressed concerns about the lack of follow-up care after the diagnostic process. For some, services were limited to a prescription for medication without additional support or follow-up, leaving them with many unanswered questions about their ADHD diagnosis and a limited understanding of how to cope. The majority of young adults emphasized the importance of receiving appropriate information and follow-up care after being diagnosed with ADHD. They expressed a desire for referrals to (informal) networks or other accessible care options (quote 24–26).

What I also think is important is that as soon as there is a diagnosis, there is some kind of action plan and a protocol. . . . Where do you find peers . . . . You indeed feel so alone that you think ‘what is wrong with me?’ At that moment you really feel like the only one who has it [ADHD] and you just want to talk about it. Maybe someone has a tip that you can also benefit from . . . . That it doesn’t stop at ‘okay you got it [ADHD], we’ll put you on Ritalin, see how that goes and if it goes well, good for you, if not, good luck with it’. . . . My self-concept really went down as a result, because I didn’t know how to deal with it. (Q24, R1, Women FGD 1)

Mental health care professionals also highlighted a lack of continuity of care for individuals with ADHD which hampered access to care. This included the lack of follow-up over time to assess whether individuals still meet diagnostic criteria. For example, after the diagnostic trajectory and set up of the medication treatment, clients are referred back to the GP. Should they encounter new challenges that require specialized ADHD care at a later stage, they are placed back on typically long waiting lists, posing an additional barrier to accessibility. Consequently, individuals tend to seek assistance only when their symptoms or challenges have escalated (quote 27). The division of ADHD care between children (<18 years) and adults (>18 years) further disrupts continuity of care. Upon turning 18 years old, clients need to be referred to a new health care provider by their GP and often face another waiting list.

Lastly, young adults and parents expressed the need for peer support, wherein “peer” denotes individuals with lived experiences. Some of the young adult women conveyed they gained valuable insights from peers with ADHD. At the same time, some women indicated the scarcity of individuals with ADHD within their social circles (quote 29). This highlights the need for accessible peer support networks. Young adults and parents acknowledged to experience validation, a sense of community, and practical advice through peer interactions (quote 28–31).

R8: “What I think we need is more community. . . Finding others has been very helpful to me. I’ve actually learned as much, if not more, from other people that I know have ADHD . . . other than from my therapy and that’s because you can just very practically exchange things with each other . . ., together you can broaden the picture of what ADHD is.”

R6: “But I don’t actually know very many people who have it.” (Q29, R8 & R6, Women FGD 2)

## Discussion

In this qualitative study aimed to identify unmet needs of children and young adults with ADHD in the context of ADHD-related stigma reduction in the Netherlands, three key thematic areas emerged: (1) awareness, knowledge, and understanding of ADHD; (2) personalized approaches in education and health care; and (3) accessibility of information and support services. Subsequently, each of these themes encompassed specific unmet needs identified by respondents across all stakeholder groups. We found persistent limited understanding of ADHD as a multifaceted biopsychosocial condition, which manifested in the cognitive, affective, and behavioral responses toward children and young adults with ADHD as reported by key stakeholder groups.

Our findings are in line with previous research that highlights limited knowledge and diverse understandings of ADHD. This includes studies involving various stakeholders (see Carr-Fanning, 2023; van Langen et al., 2023), consensus statements (see Kooij et al., 2019; Faraone et al., 2021), and literature reviews (see Bisset et al., 2021, 2023; Mueller et al., 2012). This can translate to different views regarding how to support and include individuals with ADHD. Specifically, the limited awareness, knowledge, and understanding of ADHD (theme 1) directly influences the reported unmet needs in education and health care services highlighted under theme 2 and 3. In education, stakeholder groups in our study highlighted inappropriate and even harmful accommodations (e.g., extra time during tests) or discrimination (e.g., exclusion from the classroom). These findings align with findings from Baeyens (2021), who substantiates the limited effectiveness of certain accommodations for ADHD in higher education, including additional time for tests and separate room testing. In mental health care, stakeholder groups in our study reported that the societal and relational issues as well as the strengths associated with ADHD (e.g., “what does it mean to be a neurodivergent person” or how to navigate relationships at work or school) received relatively little attention as compared to symptoms and medication treatment options. Our findings are in line with findings from a previous qualitative study in the Netherlands, in which adults with ADHD reported dissatisfaction with mental health services due to being symptom-centered and lacking strength-based approaches (Schrevel, Dedding, & Broerse, 2016). Further, young adults expressed difficulties with accessing mental health care services and noted a lack of follow-up care after an ADHD diagnosis. Our study shows that while interventions exist, some are considered inappropriate or even harmful. This highlights the importance of considering ADHD as a complex biopsychosocial condition and engaging the perspectives of those with lived experiences when designing support and stigma reduction strategies.

Our findings suggest priorities for strengthening support and stigma reduction. Stakeholder groups suggested various approaches that employ some form of education to address the lack of awareness, knowledge, and understanding of ADHD (theme 1 and 2). Respondents specifically suggested to improve education for teachers and (mental) health care professionals, and advocated for the integration of more personalized approaches, as well as education on neurodiversity, across both lower and higher education settings. The recommendation to focus on awareness and education corresponds with conclusions from recent qualitative research on ADHD including various stakeholders in Ireland and the Netherlands (Carr-Fanning, 2023; van Langen et al., 2023). Simultaneously, the young adults in our study expressed concern that an excessive focus on ADHD or neurodiversity in education could be harmful, as it may lead to special treatment and unnecessarily highlight differences. This perspective aligns with the findings of van Langen et al. (2023), who identified dormant ambivalence in stakeholders’ perspectives on ADHD. They for instance found that considering ADHD as a category was perceived to be both helpful and harmful. The need for education about ADHD as well as neurodiversity reflects a common approach employed in stigma reduction literature. Previous research on mental health service users’ experiences with discrimination suggests that education needs to be targeted to group-specific contexts and behaviors to be effective (Hamilton et al., 2016). However, reviews on stigma reduction approaches have been critical toward the stigma reducing potential of educational approaches or interventions focused only on mental health literacy, given the lack of conclusive evidence regarding their efficacy for changing behaviors (Heijnders & van der Meij, 2006; Rüsch et al., 2005; Stuart, 2016). Corrigan (2016) even emphasized the risks associated with educating about mental health problems, highlighting the potential for unintended negative impacts. This includes the risk of endorsing the view that a potential disorder is solely due to brain or genetic dysfunction, which can perpetuate beliefs that individuals are incompetent or unable to recover. For example, a study examining the impact of education endorsing biological and psychosocial explanations of ADHD revealed that while biological explanations can mitigate social rejection, they may also cultivate beliefs that ADHD is untreatable (Lebowitz et al., 2016). Education should thus be best regarded as an initial step in stigma reduction and shows promise when integrated with other strategies, such as contact with people with lived experiences (Corrigan et al., 2012; McCullock & Scrivano, 2023).

Another important finding concerns a priority for mental health care services, as respondents suggested to facilitate engagement with peers within support and health services and to improve the accessibility of services. Previous meta-analyses have evaluated the impact of peer support or peer-led interventions in mental health and revealed moderately positive outcomes on various dimensions of stigma such as self-stigma, stigma related stress or empowerment (Burke et al., 2018; Sun et al., 2022). Our findings align with a previous trend in research, as illustrated by a 2016 commentary, that identified peer support as a valuable intervention in mental health care and highlighted the need for further assessment (Naslund et al., 2016). Moreover, a 2023 report on mental health services in the Netherlands echoed the need for accessible peer support and mental health care services, without the need to enter a formal care trajectory, as well as for care tailored to individual needs and social interactions (Boumans et al., 2023).

Lastly, our study highlights changes that should be initiated at a structural or organizational level of education and health care institutes (e.g., improving education, increasing capacity for personalized approaches, accessible services) in order to address stigma at the individual and interpersonal socioecological level. Although stakeholder groups in our study generally agreed on thematic categories of priority to improve support for individuals with ADHD, their perspectives on how to actualize change in certain settings seemed to vary. For example, young adults mentioned that education should aim for changing norms, values and practices and should by design suit neurodiversity to facilitate inclusive environments. While teachers agreed with these goals, they referred to challenges in terms of individual and school capacity to accommodate this. Two previous studies from an Irish context have also found this perspective (Carr-Fanning, 2023; Shevlin et al., 2013). Shevlin et al. (2013) reported that, based on qualitative interviews with school principals, teachers, and support staff, there was generally a supportive attitude toward facilitating inclusion. However, there were concerns about the individual and organizational capacity to establish these inclusive environments.

The unmet needs and priorities for stigma reduction found in this study have several implications for future research and stigma reduction efforts. Our findings are consistent with previous research that highlights the need for multi-level interventions. As such, while increasing mental health literacy and raising awareness of neurodiversity are crucial, these strategies alone may be insufficient to effectively reduce the negative stereotyping, prejudice, and discrimination associated with ADHD. The unmet needs identified in this study are closely linked to the responsibilities of various actors at the organizational and structural level, such as teachers and (mental) health professionals, as well as to the accessibility and quality of support and health services needed to cultivate inclusive practices. Furthermore, efforts to reduce stigma should involve collaboration with individuals who have lived experiences, with support from educators and mental health care professionals, in order to address group-specific needs (Ashton et al., 2018). Contact with people with lived experiences has been found most effective in reducing mental health stigma, and people with lived experiences are considered important change agents (Thornicroft et al., 2022). While stigma reduction strategies involving education and contact have presented promising results, more research is warranted to assess these strategies, and few studies have explored their long-term impact on attitudes or behavior change (Dalky, 2012; Gronholm et al., 2017; Jorm, 2020; A. J. Morgan et al., 2018). Moreover, future (participatory) research is needed to determine and develop relevant strategies for supporting individuals with ADHD and to investigate the efficacy of various efforts, such as peer support.

Despite the various strengths, there are some limitations to the study of note. Due to the low sample size per stakeholder group, we prioritized young adults perspectives to identify key themes and we were not able to compare responses between stakeholder groups and determine a hierarchy to priorities across stakeholders. However, while unmet needs differed slightly between stakeholder groups as some related to context-specific professional perspectives, respondents generally agreed on the presented thematic areas of priority for stigma reduction. Furthermore, due to the (relatively small) convenience sample lacking diversity in terms of ethnicity and education levels, our ability to capture a comprehensive range of perspectives and experiences related to ADHD-related stigma was limited. On the other hand, given our choice to organize small FGDs, we were able to collect rich and detailed respondent perspectives on unmet needs and priorities for change. Lastly, the Dutch context is important to consider when interpreting our results. The Netherlands is a high-income Western European country, with a well-organized health care system, including standards and specialist care for ADHD. A recent literature review on unmet needs of individuals with ADHD and their caregivers, encompassing 23 studies from Australia, Canada, various European countries, Hong Kong, Iran, and the U.S, identified highly similar unmet needs to those identified in our study (Bisset et al., 2023). This suggests that our findings may be relevant beyond the Dutch context. However, there is still a notable scarcity of research from lower-income countries and contexts where ADHD receives limited attention. Future studies should address unmet needs in other geographical regions, countries, and demographics, with particular attention to explore the role of other intersecting identity markers (e.g., gender, socioeconomic status, ethnicity, and race; see for example Bergey et al., 2022).

This paper highlights three significant themes pertaining to the unmet needs of children and young adults with ADHD for stigma reduction. It is imperative for efforts aimed at reducing ADHD-related stigma to recognize ADHD as a complex biopsychosocial condition. Given the relatively limited attention to date, it is important to address the psychosocial factors that influence the (mental) health and well-being of individuals with ADHD. For example, by designing education to suit neurodiversity and including strength-based approaches and addressing relationships at work or at school in mental health care. This qualitative study underscores the importance of targeted multi-level efforts, and reveals that such efforts to disrupt stigmatization should prioritize improving societal awareness, knowledge, and understanding of ADHD and neurodiversity, particularly among primary school teachers and (mental) health care professionals. Finally, the personalization and accessibility of health and support services are emphasized as crucial for effectively addressing ADHD-related stigma.

## Supplemental Material

sj-docx-1-jad-10.1177_10870547241297876 – Supplemental material for Unmet Needs of Children and Young Adults With ADHD: Insights From Key Stakeholders on Priorities for Stigma Reduction Supplemental material, sj-docx-1-jad-10.1177_10870547241297876 for Unmet Needs of Children and Young Adults With ADHD: Insights From Key Stakeholders on Priorities for Stigma Reduction by Marlies Jolinde Visser, Ruth Maria Hendrika Peters and Marjolein Luman in Journal of Attention Disorders

sj-docx-2-jad-10.1177_10870547241297876 – Supplemental material for Unmet Needs of Children and Young Adults With ADHD: Insights From Key Stakeholders on Priorities for Stigma Reduction Supplemental material, sj-docx-2-jad-10.1177_10870547241297876 for Unmet Needs of Children and Young Adults With ADHD: Insights From Key Stakeholders on Priorities for Stigma Reduction by Marlies Jolinde Visser, Ruth Maria Hendrika Peters and Marjolein Luman in Journal of Attention Disorders

## Notes

1. PABO is the name of the training program to become a primary school teacher in the Netherlands.
