# Supplementary material for: Unmet Needs of Children and Young Adults With ADHD: Insights From Key Stakeholders on Priorities for Stigma Reduction
Source: J Atten Disord. 2024 Nov 15;29(3):195–206. doi: 10.1177/10870547241297876 (PMC11694544; doi:10.1177/10870547241297876)
Supplement: sj-docx-1-jad-10.1177_10870547241297876 – Supplemental material for Unmet Needs of Children and Young Adults With ADHD: Insights From Key Stakeholders on Priorities for Stigma Reduction [file sj-docx-1-jad-10.1177_10870547241297876.docx]

**Supplementary Material 1**

**Table S1**

*Design of Activity Three of the Focus Group Discussion with Young Adults*

| **Structure** | **Description and key guiding questions** | **Format** |
| --- | --- | --- |
| *Activity 1 and 2 are beyond the scope of this paper* | | |
| Activity 3. Stigma reduction | Question 1: How do you see the role of (individuals with ADHD, family, teachers, healthcare providers, employers, the social environment, and society) in reducing negative associations and prejudices related to ADHD and in strengthening positive associations?   - What support would be needed for this?   Question 2: What could others (healthcare providers, teachers, family, social contacts, society) do to better support and include people with ADHD? Do you have personal experiences with actions that have helped you?   - During school or education at a younger age - During education, studies, or work as an adult   Question 3: What would help to protect or promote the self-esteem of people with ADHD?   - At a younger age - As an adult | Phase 1. Individual writing on sticky notes  Phase 2. Group discussion |
| Wrap-up | Question 4: Has everything been discussed, or are there any additional things you’d like to share or discuss?  Thanking respondents for their effort and time. | Group discussion |
