# Supplementary material for: Unmet Needs of Children and Young Adults With ADHD: Insights From Key Stakeholders on Priorities for Stigma Reduction
Source: J Atten Disord. 2024 Nov 15;29(3):195–206. doi: 10.1177/10870547241297876 (PMC11694544; doi:10.1177/10870547241297876)
Supplement: sj-docx-2-jad-10.1177_10870547241297876 – Supplemental material for Unmet Needs of Children and Young Adults With ADHD: Insights From Key Stakeholders on Priorities for Stigma Reduction [file sj-docx-2-jad-10.1177_10870547241297876.docx]

**Supplementary Material 2**

**Table S2**

*Key Supporting Quotes*

|  | **Dutch quote** | **English translation** |
| --- | --- | --- |
| **1** | R14: “Voor mijn gevoel wordt er altijd heel erg op de concrete problemen gefocust. Als het erop aankomt dan moet je maar gewoon stil zitten en opletten en het werk doen. Dan is het ‘druk zijn’ nog steeds een probleem en de voordelen is dan vooral maar iets leuks. Maar eigenlijk wordt er nog steeds gedaan van, de symptomen moeten behandeld worden om in ‘de normale samenleving’ te kunnen passen. Terwijl dat volgens mij helemaal niet de manier is om met de gezondheid van de mensen met ADHD voorop een behandeling op te zetten. Ik heb het idee dat het veel meer zou helpen als het beeld van ADHD … zou veranderen naar iets wat voor meer ook emotionele zelfregulatie en het vermogen om jezelf te begrijpen en, ja begrip van tijd en van relaties met anderen, dat dat voor mij veel meer de challenges zijn geweest.”  (Men FGD 2) | “I feel that the focus has always very much been on the concrete problems. When it comes down to it, you just have to sit still and pay attention and do the work. Then 'being hyperactive' is still a problem and the benefits are mainly something nice to have. But actually, it is still the case that the symptoms have to be treated in order to fit into 'normal society'. While in my opinion that is not at all the way to set up a treatment considering the health of people with ADHD first. I feel it would be much more helpful if the picture of ADHD … changed to something that includes more emotional self-regulation and the ability to understand yourself and, yes, understanding time and relationships with others, that for me have been much relevant challenges.” |
| **2** | R8: “Dat het erkend wordt dat we anders zijn, maar niet per se slechter of niet meedraaien met de maatschappij. Dus ik zou het heel fijn vinden als er inderdaad een punt op je werk komt of op school waar je om hulp kan vragen. Maar er zit eigenlijk ook de aanname in dat er iets aan jou ontbreekt en dat je iets extra’s nodig hebt, terwijl wat ik juist ook heel fijn zou vinden is als er meer naar de positieve kanten van ADHD wordt gekeken. … Ik zou het heel leuk vinden als we met z’n allen op een andere manier naar kennis en naar leren kijken. De reden dat we denk ik moeite hebben met meedraaien, heeft heel veel te maken met dat wij op een andere manier leren of met de wereld omgaan dan heel veel mensen, want we denken heel associatief. Maar tegelijkertijd is dat juist heel erg waardevol en moeten we dat juist meer omarmen.”  (Women FGD 2) | “That it is recognised that we are different, but not necessarily in a worse way or that we are not keeping up with society. So, I would really like it if there would be a point at work or at school where you can ask for help. But there is actually also the assumption that you lack something and that you need something extra, while what I would really like is if more attention is paid to the positive sides of ADHD. … I would really like it if we all look at knowledge and learning in a different way. I think the reason we have trouble keeping up has a lot to do with the fact that we learn or deal with the world in a different way as compared to a lot of people, because we think very associatively. But at the same time that is very valuable, and we should embrace it more.” |
| **3** | R2: “Ik denk eigenlijk dat het, het meeste neerkomt op het thema onderwijs. Maar ik denk met name dat, ik denk dat het breder geldt, het omarmen van diversiteit in de breedste zin van het woord. Dat dat heel mooi zou zijn. Bijvoorbeeld in het onderwijs dat er meer aandacht komt voor neurodiversiteit, diversiteit daarmee ook neerzetten als een kracht en daarmee ook meer vanuit de maatschappij gaan kijken van ‘oké, hoe kunnen we vanuit een positieve invalshoek deze mensen inzetten en wat hebben ze te bieden?’ Meer ruimte voor de vrije talentontwikkeling.” (Women, FGD 1) | “I actually think it comes down to the theme of education specifically. I think in particular that, I think it applies more broadly, embracing diversity in the broadest sense of the word. That that would be really nice. For example, in education that more attention is paid to neurodiversity, thereby also establishing diversity as a strength and thereby also looking more from a societal perspective, such as 'okay, how can we apply these people from a positive angle and what do they have to offer?' More space for free talent development.” |
| **4** | R9: “Ik denk dat er kansen zijn om het juist op een breed en subtiel aan te kaarten. Ik bedoel we moeten niet nu met een grote stempel neurodiversiteit gaan aankondigen. Dan schiet het precies het doel voorbij… dan krijg je weer die speciale behandeling. Nee, maar een stukje bewustwording. … Ik zit te denken aan lessen waarbij … jij en je medestudenten erachter komen hoe je bepaalde dingen aanpakt. Dus hoe je opdrachten maakt, … zonder waardeoordeel. Dat je van elkaar ontdekt hoe verschillend je eigenlijk bent, en dat je elkaar kan respecteren.”  (Men, FGD 1) | “I think there are opportunities to address it in a broad and subtle way. I mean we shouldn't announce neurodiversity with great emphasis now. Then it misses the mark… then you get that special treatment again. No, just some awareness. … I'm thinking of lessons [in school] where… you and your fellow students find out how to approach certain things. So how to make assignments, ... without value judgment. That you discover how different you actually are from each other, and that you can respect each other.” |
| **5** | R17: “Dat is ook het probleem want er bestaat een stereotiep van ADHD wat vooral gebaseerd is op de negatieve afwijkingen in gedrag en daar ligt vaak de focus op. In mijn beleving is dat ook vaak wat je ook terugkrijgt als ouder zijnde van goh er is iets met je kind aan de hand want ze is altijd zo druk …. Docenten in vergaderingen zeggen van ja weet je, dit kind vertoont dit en dit gedrag, dat hoort niet in deze klas met dit gedrag. En daar is volgens mij heel veel winst te behalen. Omdat dat negatieve nog altijd de boventoon voert. En de andere kanten, de positieve kanten van ADHD, het druk zijn betekent ook dat iemand, ja, misschien wel heel goed mee wil denken, of heel enthousiast is. En, en als je altijd vragen stelt ben je misschien wel gewoon heel nieuwsgierig.”  (Parents) | “That is also the problem because there is a stereotype of ADHD that is mainly based on the negative deviations in behaviour and that is often what is focused on. In my experience, that is often what you also get back as a parent like ‘gosh there is something wrong with your child because she is always so hyperactive’…. Teachers in meetings say yes, you know, this child exhibits this and this behaviour, that does not belong in this class with this behaviour. And I think there is a lot to be improved there. Because that negativity still predominates. And the other sides, the positive sides of ADHD, being hyperactive also means that someone maybe wants to engage or is very enthusiastic. And if you always ask questions, you might just be very curious.” |
| **6** | R6: “Ze [leraren] weten ook niet hoe ze je kunnen helpen, dat ook wel. Ze weten misschien wel dat je het hebt, maar dan kunnen ze er verder niet heel veel mee, dan denken ze nou ja, laat maar.”  (Women, FGD 2) | “They [teachers] don't know how to help you, that's for sure. They may know that you have it [ADHD], but then they can't do much with it, so they think, well, never mind." |
| **7** | R3: “Onder collega’s die werken met kinderen en jongeren is daar wel een oké beeld… Vaak van ‘ja kunnen we die diagnose wel stellen, want er is ook trauma. Dus … die ADHD daar kunnen we ons ook niet op richten’. En dan gaan ze helemaal erna voorbij van het beschrijven van de symptomen. Bij de volwassenen vind ik echt…. als we discussie hebben van waar moet geld naar gaan waar moet ruimte voor zijn dan gaat onze 18-jarige krijgt in ons eigen ziekenhuis niet bij de volwassen psychiaters omdat er geen plek is.”  I: “Ja en leidt dus dat beeld dat het minder belangrijk is leidt dat dus in jouw ervering ook naar minder diagnose?”  R3: “Ja denk ik wel dat mensen die zich pas later presenteren die dan zich pas de vraag stellen of dan pas in moeilijkheden komen. Ik had gisteren een gesprek met een psychiater van daar en die doet ook de verslavingszorg en ja er komen daar ook mensen met zo een duidelijk ADHD-beeld is die dan ook een verslaving ontwikkeld hebben en dan gaat iedereen zich richten op de verslaving en niemand op ADHD en dan lijkt verslaving het primaire probleem.”  (Mental health professionals) | R3: “Among colleagues who work with children and young people, there is an okay perception of this.... Often the case that 'yes, we can make that diagnosis, because there is also trauma. So, then we can't focus on ADHD.' And then they completely ignore describing the symptoms. With adults, I really think that when we have a discussion about where money should go to what should there be room for, then our 18-year-old does not go to the adult psychiatrists in our own hospital because there is no room.”  I: “Yes, and does that image that it is less important also lead to less diagnosis in your experience?”  R3: “Yes, I think that people who present themselves later only then ask themselves the question or only then encounter difficulties. Yesterday I had a conversation with a psychiatrist from there and he also does addiction care and yes, there are also people there with a clear ADHD picture who have also developed an addiction and then everyone focuses on the addiction and no one on ADHD and then addiction seems to be the primary problem.” |
| **8** | R21: “Het is ook een beetje … dat zwart-witte: je weet wel veel van ADHD of je weet er niks van. Dus sommige mensen houden zich er dan ook heel erg van afzijdig, omdat ze er eigenlijk niet zoveel vanaf weten. Terwijl het eigenlijk gewoon iets is waar je gewoon als behandelaar de kennis van moet hebben”  (Mental health professionals) | “It's also a bit black and white: you either know a lot about ADHD or you know nothing about it. So, some people really shy away from it, because they don't really know much about it. While it is actually something that you as a practitioner simply need to have knowledge of.” |
| **9** | R19: “Wij hebben dus geen psychiater meer in huis en dan is het ook echt moeilijk om die medicatie gemonitord te krijgen en dat je dan dus ziet dat ze zeggen van ‘nee, de medicatie is gewoon goed daar ligt het niet aan maar toch loopt het niet’ en dat het dan ook best moeilijk is om daar weer het gesprek over aan te gaan van ja als dit kind inmiddels 16 is en op 12 jaar voor het laatst is ingesteld, ja dat daar dus ook bij psychologen weinig kennis over is dat dat dus heel belangrijk is om te monitoren en opnieuw te laten instellen"  (Mental health professionals) | “We no longer have an in-house psychiatrist and then it is really difficult to have the medication monitored and you see that they [psychologists] say 'no, the medication is just fine, that is not the reason, but still it is not working’ and that it is therefore quite difficult to start the conversation about this again: yes, if this child is now 16 and the last setting was at the age of 12, yes, that means that psychologists also have little knowledge about this. is very important to monitor and have it reset." |
| **10** | R14: “Als je het begreep, dan lukte het wel. Dat vind ik dan interessant, omdat ik dan niet per se zo druk was, en omdat het op zich wel ging, ik haalde gewoon mijn punten. Het was nooit echt serieus genomen door leraren, daarom heb ik het zelf ook nooit echt serieus ben gaan nemen. Ook omdat ik het idee heb dat mensen denken dat je je gewoon aan het aanstellen bent. Daar heb ik het verder ook op de middelbare school helemaal nooit heb om er mee te dealen. Ik kreeg medicatie, maar ik heb nooit echt bij mezelf erbij stil gestaan heb dat ik dacht wat voor verdere gevolgen kan dit nog hebben voor de rest van mijn leven. En dat heef juist heel veel invloed gehad op toen ik later naar de universiteit ging. Ik ging natuurkunde studeren. Dan kan je wel goed zijn in wiskunde op de middelbare school, maar … dan moet je wel echt studeren. Daar doen ze het hele middelbare school wiskunde even in twee weken herhalen. Toen liep ik helemaal vast, direct. Ik had echt zoiets van wat is dit, ik moet een boek opendoen. Dat gaat helemaal niet. Ik heb dus nooit echt de skills geleerd, of ben gemotiveerd om te gaan met de ADHD. Ook natuurlijk omdat het allemaal wel ging, en ik was ook niet zo druk. Dat was wel vervelend. Daar heb ik wel lang last van gehad. Liever was ik eerder gaan leren om er mee om te gaan.” | “If you understood it, you could do it. I find that interesting, because I wasn't necessarily that hyperactive, and because it went well, I just got my points. It was never really taken seriously by teachers, which is why I never really started taking it seriously myself. Also because I have the feeling that people think you are just seeking attention. I never had to deal with that at all in high school. I was given medication, but I never really thought about what further consequences this could have for the rest of my life. And that had a great influence on when I later went to university. I went to study physics. Then you can be good at math in high school, but... then you really must study. There they repeat all high school math in two weeks. Then I got completely stuck, immediately. I was really like what is this, I must open a book. That doesn't work at all. So, I never really learned the skills or was motivated to deal with my ADHD. Also, of course, because everything went well, and I wasn't that hyperactive either. That was quite annoying. I struggled with that for a long time, I would rather have learned to deal with it sooner.” |
| **11** | R2: “…je krijgt… langer voor een toets, dan plak je een stempel van jij bent anders en jij krijgt iets. Op het moment dat je…het vanuit een kracht gaat benaderen, vanuit een beeld van diversiteit, dat zou mij echt al een heel ander gevoel mee hebben gegeven als kind.”  (Women FGD 1) | “…you get… more time for a test, then you label someone, like you are different, and you get something. … The moment you start approaching it as being a strength, from a diversity perspective, that would really have given a completely different feeling to me as a child.” |
| **12** | R15: “Mijn dochter en mijn middelste zoon zijn allebei heel erg intelligent. En dan zie je eigenlijk wat een verschil er tussen docent is. Die eenduidigheid is zo belangrijk bij kinderen, en het is elk jaar een zoektocht. En het ergste is nog omdat als zij dan goed op medicatie waren ingesteld kwamen ze echt wel lekker mee en dan kwam je op school en dan vraag je, ‘hoe gaat het met mijn kind’ en dan zeggen ze, ‘er is nooit wat met jou kind’. … Ja ik heb daar denk ik wel echt de meeste energie op verloren, dat je echt denkt van ja het is niet aan de school om de diagnose van mijn kinderen ter discussie te stellen. En ook een kind dat goed kan leren heeft recht op informatie.”  R16: “Ja dat merk ik ook wel hoor. Uhm, mijn zoon heeft twee verschillende docenten en bij de een heb ik … het idee dat ze het een beetje bagatelliseert, dat ze zoiets heeft van pff ja, hij doet het goed op school waar maak je je druk om. En de andere docent neemt het heel erg serieus en gaat ook samen met hem zitten en zorgt ook ervoor dat hij extra tijd krijgt bij toetsen, dat hij toch uitgedaagd wordt bij de vakken waar … het gewoon vanzelf gaat, wat meer aandacht bij de vakken die wat minder goed gaan. Dus daar zit daar ook echt een heel groot verschil tussen.”  (Parents) | R15: “My daughter and my middle son are both very intelligent. And then you see what a difference there is between teachers. That clarity is so important with children, and it is a challenge every year. And the worst part is because if they were well adjusted to medication, they really did well in school and then you come to school and ask, ‘how is my child doing’ and then they say, 'there is never anything with your child'. … Yes, I think I really lost most of my energy on that, that you really think yes, it is not up to the school to question the diagnosis of my children. And a child who can study well also has a right to information.”  R16: “Yes, I notice that too. Uhm, my son has two different teachers and with one of them … I have the feeling that she thinks like ‘pff yes, he is doing well at school why do you worry’. And the other teacher takes it very seriously and also sits down with him and ensures that he gets extra time for tests, that he is still challenged in the subjects where … he is doing well, a little more attention to the subjects that are going less well. So, there is a really big difference between them.” |
| **13** | R12: “Ik bedoel dat je gewoon iemand bent die gewoon druk is en niet kan opletten. Dat is meestal wat mensen denken van mensen met ADHD. … Ik had bijvoorbeeld een tafeltje, in de teamkamer. Waar ik dan elke dag mocht zitten. In plaats van dat ze gingen proberen om mij in de klas te laten zitten. Moest ik al apart in een kamertje gaan zitten. Ik weet niet of dat de slimste manier is om met ADHD’ers om te gaan. … Uiteindelijk had ik gewoon een tafeltje voor mij die dan in de teamkamer stond, met een paar spullen van mij die ook daar lagen omdat ik er zo vaak zat. Dan dachten ze denk ik ‘dan kan ie beter daar zitten, kan ie ook niemand afleiden’. Ze duwde me gewoon weg.”  (Men FGD 2) | “That you're just someone who's just hyperactive and can't pay attention. That's usually what people think of people with ADHD. … For example, I had a desk in the team room. Where I could sit every day. Instead of trying to make me sit in class. I had to sit alone in a room. I don't know if that's the smartest way to deal with people with ADHD. … In the end I just had a desk that was in the team room, with a few of my things that were also there because I was there so often. Then they thought 'he better sit there, he can't distract anyone that way’. They just pushed me away." |
| **14** | I: “Is daar de ruimte voor [voor differentiatie] in het onderwijs of is dat er niet?”  R23: “Ik denk weinig hoor.”  R22: “Nog onvoldoende.”  R23: “Want je hebt zoveel doelen die behaald moeten worden, zoveel lessen die afgestreept moeten worden, waar echt altijd te weinig tijd is…”  R22: “Misschien is dat ook niet zozeer alleen de taak van de leerkracht, tuurlijk moet de leerkracht ook een band op gaan bouwen […]. Ik zou eigenlijk ook wel willen dat een specialist een kind met ADHD uit de klas neemt en zegt van “joh vertel eens hoe gaat het in de klas? Stel dat het even niet gaat, wat zoek je dan op in de klas? Geef je dat aan? Wat heb je nodig om je te concentreren? Werkt een koptelefoon of…” Op die manier begeleiding krijgen, nu is het eigenlijk gewoon een kind met ADHD gaat gewoon met de diagnose in de klas zitten, wel of geen medicatie, en de leerkracht moet zonder achtergrond, zonder extra training, zonder iets van de PABO, moet gaan ontdekken van hoe ga je met zo’n leerling met ADHD om. Kan je dat van een leerkracht verwachten met 29 anderen? Is daar niet gewoon echt een specialist voor nodig die een kind daar ook in helpt?”  (Teachers) | I: “Is there room for this [differentiation] in education?”  R23: “I don't think much.”  R23: “Still insufficient.”  R23: “Because you have so many goals that need to be achieved, so many lessons that need to be crossed off, and there is never enough time…”  R22: “Perhaps that is not so much the task of the teacher alone, of course the teacher also has to build a bond […]. I would actually also like a specialist to take a child with ADHD out of the classroom and say, “Hey, tell me how are things going in class? Suppose things are not going well; what do you look for in class? How can you share this? What do you need to concentrate? Getting guidance in that way, now it's really just a child with ADHD just goes to class with the diagnosis, whether or not medication, and the teacher has to discover without background, without extra training, without anything from the PABO how you deal with such a student with ADHD. Can you expect that from a teacher with 29 others? Doesn't that actually require a specialist who also helps a child with that?" |
| **15** | R2: “In alle eerlijkheid, buiten de herkenning die je eruit haalt, wat heel fijn is, heb ik er praktisch niks aan gehad. Dat is dan wel pijnlijk. […]. Ik had dus wel best wel een herkenning gelukkig met m’n groep. Ik voelde me niet begrepen door de hulpverlening.” (Women FGD 1) | “In all honesty, apart from the recognition you get out of it, which is very nice, practically I did not get anything out of it [group therapy]. That is painful. […]. So, I felt recognised, fortunately, within my group. But I did not feel understood by the health providers.” |
| **16** | R11: “[Ik had graag] meer gesprekken [gehad] die wat dieper gaan ook over de persoon die je zelf bent denk ik. En bij de GGZ was alles heel oppervlakkig, en moest je alles dan maar heel oppervlakkig een beetje met elkaar het bespreken.”  (Men FGD 1) | “[I would have liked to have] more conversations that go a little deeper, also about the person you are. And with the GGZ everything was very superficial, and you only had to discuss everything very superficially with each other.” |
| **17** | R8: “Ik miste heel erg dat gemeenschappelijke aspect of dat sociale aspect van ADHD in mijn therapie. Niet dat therapie slecht was, ik heb er echt heel veel aan gehad, maar het ging heel erg over mij als individu en wat ik kan verbeteren. Ikzelf ben overtuigd van het feit dat het niet alleen maar een stoornis is, maar ook iets is in relatie tot de maatschappij. Ik mis dan juist eigenlijk heel erg het gesprek over “wat betekent het nou om een neurodivergente persoon te zijn in die wereld, als groep, maar ook ADHD’ers in die wereld.” We leren heel veel van elkaar, en ik denk dat neurodivergente mensen heel veel van elkaar kunnen leren en het ook nodig hebben. Juist ook heel veel van de problemen waar wij tegen aanlopen dat die maatschappelijk zijn gedefinieerd. En dat voor mij, omdat ik dus zelf contact heb gelegd met andere mensen die ook ADHD hebben, heb ik daar heel veel zingeving in gevonden, omdat je, ja je voelt je toch een beetje alleen in je therapie, als je dat hebt gehad.”  (Women FGD 2) | “I really missed that community aspect or that social aspect of ADHD in my therapy. Not that therapy was bad, it really helped me a lot, but it was very much about me as an individual and what I can improve. I myself am convinced that it is not just a disorder, but also something in relation to society. I really miss the conversation about "what does it mean to be a neurodivergent person who is in that world, as a group, but also ADHD people in that world.” We learn a lot from each other, and I think neurodivergent people can learn a lot from each other and need it. In fact, many of the problems we encounter are socially defined. And that for me, because I have made contact with other people who also have ADHD, I have found a lot of meaning in that, because you, yes, you feel a bit alone in your therapy, if you have had that.” |
| **18** | R13: “…Ook van het beeld van ADHD is gewoon een druk mens. Maar je voelt je alleen en dan ook nog de duizend andere dingen. Dan ga je denken is er nog meer mis met me.  R12: Ik kreeg ook wel echt toen hoe dat ging met de medicatie. Na een tijdje was ik er klaar mee, van nee wat? Ik mag niet mezelf zijn. En dat vond ik gewoon, nou heel fout eigenlijk. Dat ik met dat gevoel zat, van deze diagnose moet me toch gewoon helpen. Want die medicatie moet jouw probleem niet oplossen, maar het moet oplossen dat jij problematisch bent.  R14: Ja, precies dat.  R13: Het lijkt alsof het meer voor anderen is dan voor jezelf.”  (Men FGD 2) | R13: “…Also the image of ADHD is just a hyperactive person. But you feel alone and then also the thousand other things. Then you start to think is there even more wrong with me.  R12: I also really got then how that went with the medication. After a while I was done with it, like ‘no what?’ I can't be myself. I just thought that was wrong. That I had that feeling, like this diagnosis should help me. Because that medication does not solve your problem, but it solves that you are problematic.  R14: Yes, exactly that.  R13: It seems like it is more for others than yourself.” |
| **19** | R11: “Vooral het goede in iemand stimuleren. Dus, iemand met ADHD positief benaderen, en wanneer iemand iets goed doet het dus ook echt aangeven. In plaats van alleen maar het negatieve benadrukken. Want als je ADHD hebt, heb je soms toch al het idee dat je anders bent dan anderen, maar als je dan juist het positieve benadrukt, dat iemand dan een goed gevoel kan krijgen. En dat is wel echt iets wat ik wel echt gemist heb. En ook vanuit docenten dat je niet als je het goed hebt gedaan, ga je gewoon door. En als je wat fout doet dat je ook echt een uitbrander krijgt, en je staat er dan ook niet echt bij stil.”  (Men FGD 1) | “Especially stimulate one’s strengths. So, approach someone with ADHD positively, and when someone does something well, really mention it. Instead of just emphasising the negative. Because if you have ADHD, you sometimes already have the idea that you are different from others, but if you emphasise the positive, then someone can get a good feeling. And that's really something I really missed. Also, from the perspective of teachers that you not just move on when you have done well, while if you do something wrong you get a scolding. Then you also don’t reflect on what happened.” |
| **20** | R3: “Benadrukken van kracht en ontwikkeling daarvan in plaats van werken aan je zwakte.” (Women FGD 1) | “Emphasising strengths and developing these, instead of working on weaknesses.” |
|  |  |  |
| **21** | R2: “In plaats van juist het wegzetten als anders, wil je [ADHD] juist meer integreren in de maatschappij.”  (Women FGD 1) | “Instead of labelling it away as different, you want to integrate [ADHD] into society” |
| **22** | R20: “Bij het grote publiek het is zo lastig is dat mensen door de bomen het bos niet meer zien. En dat er dus zoveel informatie is en dat niemand goed weet aan waar. Wat is nou echt betrouwbaar? Dat visies soms verschillen. […] Ik denk ook niet dat er per se heel veel nieuwe dingen gemaakt moeten worden, Maar dat de betrouwbare bronnen beter vindbaar moeten zijn. En dat je dus ook een plek zou willen hebben waarvan mensen weten, oké, daar kan ik naartoe.”  (Mental health professionals) | “With the general public it is so difficult that people can no longer see the wood for the trees. That there is so much information and that no one really knows where. What is really reliable? That views sometimes differ. […] I also don't think that a lot of new sources have to be made available per se. But the reliable sources must be easier to find. And that you have a place where people know, okay, I can go there.” |
| **23** | R9: “Voorkomen dat mensen zorg vermijden, en positieve benamingen gebruiken…”  R10: “Het diagnose beleid van ADHD. Voor heel veel mensen is de drempel van een huisarts naar GGZ nogal een stap. Omdat de GGZ toch een beetje gezien wordt als het overblijfsel van het gekkenhuis. …”  R11: “Ja je gaat daar natuurlijk pas heen als er echt iets mis is natuurlijk.”  R10: “…Tegelijkertijd zijn er ook al huisartsen die … mij kunnen helpen met een doorverwijzing. … Die hulp heeft dan al vaak wel een ADHD richting, maar [is] niet per se aan de hand van een diagnose. En dat maakt de drempel voor veel mensen veel lager. Iemand die bij mij op de studie zit, voor haar was dat het de reden om zich vrij te voelen om dan maar te gaan. En inmiddels is ze al wel de hele GGZ-mallemolen in, maar dat was voor haar wel echt een must ding. Het was echt een item eigenlijk; ‘wat nou als ik naar de GGZ moet’.  R11: “Sowieso dat iemand met ADHD-hulp zoekt, dat is al heel bijzonder, want wij zijn sowieso dan niet zo snel hulp vragen omdat we het eerst zelf willen uitzoeken.” (Men FGD 1) | R9: “Preventing people from avoiding care and using positive references. …  R10: “The diagnosis policy of ADHD. For many people the threshold from a GP to mental health services is quite a step. Because mental health services are kind of seen as the remnant of the madhouse. …  R11: “Yes, you only go there if something is really wrong, of course.”  R10: “At the same time there are also general practitioners who can … help me with a referral. … That help often has an ADHD direction… and [is] not necessarily based on a diagnosis. That reduces the barrier for many people. Someone who is studying with me, for her that was the reason to feel free to go. And by now she has already entered the GGZ services, but that was really a must thing for her. It was really an item actually; ‘what if I have to go to the ‘GGZ’.  R11: “In any case, that someone with ADHD seeks help, that is already very special, because we are not so quick to ask for help anyway because we want to find out for ourselves first.” |
| **24** | R1: “Wat ik ook belangrijk vind, is zodra er een diagnose is, dat er een soort plan van aanpak komt en een protocol en iets waar je ‘dit is het en we plannen je in voor een informatieronde, wat heb je nou, waar kan je tegenaan lopen’  R3: “Waar vind je lotgenoten.”  R1: “Waar vind je ook lotgenoten die hetzelfde hebben … Je voelt je dan inderdaad ook zo alleen dat je denkt “wat is er mis met mij?”, en je voelt je op dat moment ook echt de enige die het heeft en wat je gewoon wil is er ook over praten en misschien heeft iemand een tip waar jij ook weer baat bij hebt …. Dat het niet erbij blijft van ‘oké je hebt het, we zetten je aan de Ritalin, kijk maar hoe dat loopt en als het goed gaat, nou fijn voor je, zo niet, succes ermee’. Mijn zelfbeeld is daardoor ook wel echt omlaaggegaan, omdat ik niet wist hoe ik ermee om moest gaan.”  (Women FGD 1) | R1: “What I also think is important is that as soon as there is a diagnosis, there is some kind of action plan and a protocol and somewhere where you ‘this is it and we schedule you for an information round, what do you have, what are challenges’.”  R3: “Where do you find peers.”  R1: “Where do you find peers who have the same …. You indeed feel so alone that you think ‘what is wrong with me?’, and at that moment you really feel like the only one who has it [ADHD] and you just want to talk about it and maybe someone has a tip that you can also benefit from …. That it doesn't stop at 'okay you got it [ADHD], we'll put you on Ritalin, see how that goes and if it goes well, good for you, if not, good luck with it'. My self-concept really went down as a result, because I didn't know how to deal with it.” |
| **25** | R14: “Ik vind het heel stom dat ze eigenlijk gewoon zeggen, ‘hier heb je medicatie en dan moet het wel goed komen’. In plaats van dat ze er echt voor gaan zorgen dat je echt over je eigen situatie kan gaan nadenken. Want het heeft voor mij tot mijn 28ste geduurd tot ik echt dacht van dit is eigenlijk wel echt een probleem, zal ik hier eens wat mee gaan doen. En dan is het wel ja, redelijk laat om dan nog eens met iemand er over te praten. Altijd beter laat dan nooit. Maar dan moet je weer een behandelaar gaan vinden, met wachtrijen. Dat was wel veel beter geweest zijn aan het begin als iemand echt gaat, ja, toch er meer op gaat inspelen dat jij er zelf echt bewust mee bezig bent. Dat is wel iets wat ik ja heel erg gemist hebt. Daar zit ik nu wel heel erg mee.”  (Men FGD 2) | “I think it's really stupid that they actually just say, 'here you have medication and then it should work out'. Instead of really making sure that you can really think about your own situation. Because it took me until I was 28 before I really thought this is actually a real problem, I'll do something with this. And then yes, it's pretty late to talk to someone about it again. Always better late than never. But then you have to find a practitioner again, with waitlists. That would have been much better at the beginning if someone really responds more to the fact that you yourself are really consciously involved. That's something I missed a lot. I am very struggling with that now.” |
|  |  |  |
| **26** | R12: “Als je wordt gediagnostiseerd, toen dacht ik echt van ja, ik heb ADHD, en nu. Ik snapte er helemaal niks van. Door de jaren heen ben ik er langzaam achter gekomen wat het nou eigenlijk inhoudt. Maar ja, ik had ADHD. Maar ik wist niet wat ik er mee moest.”  (Men FGD 2) | “When I got diagnosed, then I really thought, yes, I have ADHD, and now what. I didn't understand it at all. Over the years I slowly found out what it actually means. But yes, I had ADHD. But I didn't know how to deal with it.” |
| **27** | R19: “Dan is dus ook de drempel om weer opnieuw aan te melden en weer op de wachtlijst gaan. Die is gewoon best wel groot, dus dan komen ze pas weer als het weer echt is misgelopen bijvoorbeeld.  R21: Ja, en dat is dus gebrek aan kennis inderdaad. Ik zeg ook altijd, ja, ik ga jullie waarschijnlijk gewoon weer terugzien over een aantal jaar, want dan heb je weer een andere, een andere setting, dan heb je weer andere problemen die zich aanbieden. Maar het stelsel is daar niet op gebouwd, dus dan kom je weer op zo'n lange wachtlijst en dan verergeren klachten weer.  R19: “Dat vind ik ook zeker lastig in die levensfase tussen zeg maar nou ja, 16 en 30 of zo. Dat ze ook wisselen, op welke plek kan ik terecht? Dat zijn dan ook echt dat het ook een drempel kan zijn om hulp te zoeken. Een levensloop traject zou je willen, waar je altijd weer terug kan komen.”  (Mental health professionals) | R19: “Then there is also the barrier to register again and go back on the waiting list. It's just quite big, so they only come again when it has really gone wrong, for example.”  R21: “Yes, and that is indeed a lack of knowledge. I always say, yes, I will probably see you again in a few years, because then you will have a different, a different setting, then you will have other problems that present themselves. But the system is not built for that, so you end up on a long waiting list again and then symptoms worsen again.”  R19: “I certainly find that difficult in that phase of life between, say, 16 and 30 or so. That they also change, where can I go? That really means that it can also be a barrier to seeking help. I would like a life cycle trajectory where you can always come back.” |
| **28** | R3: “Meer simpele manieren om onderling te verbinden, zodat het inderdaad zelf dat maatwerk, want als je dat op een heel hoog niveau inzet, zoals de DSM, dat is gegeneraliseerd, want het moet voor heel veel verschillende mensen werken en omdat neurodiversiteit zo ontzettend maatwerk is, is het veel nuttiger voor de patiënt […] om dat juist op klein niveau en juist regionaal en groepjes binnen de wijk, binnen een dorp, binnen de stad op te lossen. Ik denk dat dat hele stigma verkleind wordt als mensen zelf in hun kracht komen te staan, dus als er vanuit het grote systeem verbinding wordt gelegd naar kleinere groepen. Bijvoorbeeld, verwijzingsprotocollen voor hulpverleners als link tussen het zorgsysteem en dit soort kleinere groepen, of coaches en ervaringsdeskundigen. Ik sta nu wel goed in mijn kracht als persoon met ADHD, daardoor durf ik ervoor uit te komen, is het voor mij makkelijker, maar de mensen om mij heen leren daar heel veel van en dat normaliseert ook ADHD wat meer. Dat zorgt er ook voor dat alle mensen om mij heen die ik hierover heb gesproken, zoiets hebben van “o, zijn er zoveel verschillende dingen, het is niet alleen hyper en misschien zijn er nog andere dingen waar ik rekening mee kan houden.” Dus […] dan wordt dat stigma veel sneller verkleind omdat die informatie en die verbinding vergroot wordt.”  (Women FGD 1) | “More simple ways to connect with each other, so that it does indeed have that customisation, because if you use it at a very high level, such as the DSM, that is generalised, because it has to work for many different people and because neurodiversity is so incredibly personalised it is much more useful for the patient […] to solve this at a small level, regionally and in groups within the neighbourhood, within a village, within the city. I think that whole stigma is reduced when people empower themselves, so when connections are made from the large system to smaller groups. For example, referral protocols for care providers as a link between the healthcare system and these types of smaller groups, or coaches and experts by experience. I am empowered as a person with ADHD, that makes me dare to say it, it is easier for me, but the people around me learn a lot from it and that also normalises ADHD a bit more. That also causes all the people around me that I've talked to about this to be like ‘oh, there are so many different things, it's not just hyperactivity and maybe there are other things I can take into account’. So […] then that stigma is reduced much quicker because that information and that connection is increased.” |
| **29** | R8: “Wat ik denk dat we nodig hebben is inderdaad meer gemeenschap. […] Elkaar opzoeken, daar heb ik heel veel aan gehad. Ik heb eigenlijk net zoveel, als niet meer, geleerd van andere mensen waarvan ik weet dat ze ADHD hebben of iets anders neurodivergents dan van m’n therapie en dat komt omdat je gewoon heel praktisch dingen met elkaar kan uitwisselen van “oja, dat doe ik zo en zo en dat helpt mij” en omdat je dan samen het beeld kunt verbreden van wat ADHD is.”  R6: “Maar ik ken eigenlijk niet heel veel mensen die het hebben”  (Women FGD 2) | R8: “What I think we need is more community indeed. […] Finding others has been very helpful to me. I've actually learned as much, if not more, from other people that I know have ADHD or anything neurodivergent other than from my therapy and that's because you can just very practically exchange things with each other like "oh yeah, do that me so and so and that helps me" and because then together you can broaden the picture of what ADHD is."  R6: "But I don't actually know very many people who have it." |
| **30** | R10: “En zodra je dan je diagnose krijgt en met andere mensen met ADHD in gesprek gaat, dan kom je er ineens achter, dat het dan gewoon daarmee te maken heeft.”  R9: “Die herkenning met andere ADHD’ers.”  (Men FGD 1) | R10: “And as soon as you get your diagnosis and talk to other people with ADHD, you suddenly find out that it just has to do with that.”  R9: “That recognition with other people with ADHD.” |
| **31** | R15: [on a parent association] “Daar kun je ontzettend veel ouders ontmoeten en die volgen alle ontwikkelingen ook financieel via de overheid. Je kunt ze vragen stellen op de meest bijzondere onderwerpen en ja, ik heb daar echt heel veel steun mee gehad en nog steeds.”  (Parents) | [on a parent association] “There you can meet a lot of parents and they also follow all developments, also financial through the government. You can ask them questions on the most special topics and yes, it really gave me a lot of support and still does.” |

*Note.* GGZ is an acronym referring to public mental health care (‘Geestelijke Gezondheidszorg’) in the Netherlands.
